# MD Simulations of tRNA and Aminoacyl-tRNA Synthetases: Dynamics, Folding, Binding, and Allostery

**DOI:** 10.3390/ijms160715872

**Published:** 2015-07-13

**Authors:** Rongzhong Li, Lindsay M. Macnamara, Jessica D. Leuchter, Rebecca W. Alexander, Samuel S. Cho

**Affiliations:** 1 Departments of Physics and Computer Science, Wake Forest University, 1834 Wake Forest Road, Winston-Salem, NC 27109, USA; E-Mail: lir0@wfu.edu; 2 Department of Chemistry, Wake Forest University, 1834 Wake Forest Road, Winston-Salem, NC 27109, USA; E-Mails: lmacnama@wakehealth.edu (L.M.M.); alexanr@wfu.edu (R.W.A.); 3 Department of Physics, Wake Forest University, 1834 Wake Forest Road, Winston-Salem, NC 27109, USA; E-Mail: jessica.leuchter@gmail.com

**Keywords:** coarse-grained, atomistic, empirical force field, catalytic mechanism, editing

## Abstract

While tRNA and aminoacyl-tRNA synthetases are classes of biomolecules that have been extensively studied for decades, the finer details of how they carry out their fundamental biological functions in protein synthesis remain a challenge. Recent molecular dynamics (MD) simulations are verifying experimental observations and providing new insight that cannot be addressed from experiments alone. Throughout the review, we briefly discuss important historical events to provide a context for how far the field has progressed over the past few decades. We then review the background of tRNA molecules, aminoacyl-tRNA synthetases, and current state of the art MD simulation techniques for those who may be unfamiliar with any of those fields. Recent MD simulations of tRNA dynamics and folding and of aminoacyl-tRNA synthetase dynamics and mechanistic characterizations are discussed. We highlight the recent successes and discuss how important questions can be addressed using current MD simulations techniques. We also outline several natural next steps for computational studies of AARS:tRNA complexes.

## 1. Introduction

Crick first predicted the existence of Transfer RNA (tRNA) molecules that mediate the translation of messenger RNA (mRNA) codons into corresponding amino acids even before they were discovered [1]. The basis for his hypothesis is that while the 20 standard amino acids have great variability in sidechain types including hydrophobic, hydrophilic, charged, and aromatic moieties, nucleic acid bases form hydrogen bonds that are very good at identifying each other but not at distinguishing between different hydrophobic and hydrophilic species. He postulated that there must be “adaptor molecules” that would translate the RNA alphabet into a protein version to produce a polypeptide composed of the many different types of amino acids. In the simplest form, there would exist 20 adaptor molecules, one for each amino acid. He further posited that each of these adaptor molecules would be joined to its own amino acid by a “special enzyme”. The existence of the adaptor molecule was later confirmed [2], which we now know as tRNA, and the special enzymes are aminoacyl-tRNA synthetases.

**Figure 1:**
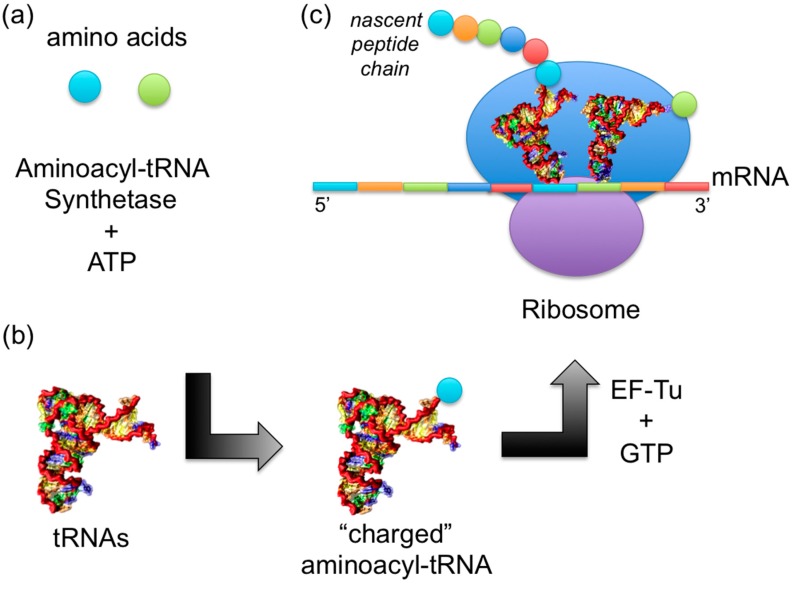
Overview of the role of tRNA in protein synthesis (**a**) Aminoacyl-tRNA synthetases catalyze the attachment of amino acids to their cognate tRNAs to result in “charged” aminoacyl-tRNAs; (**b**) Each tRNA has a specific sequence that corresponds to an amino acid. Multiple isoaccepting tRNAs may specify a single amino acid; (**c**) Nascent peptides are synthesized at the ribosome using the mRNA template. Charged tRNAs with their corresponding amino acids are delivered to the ribosome, one by one, by matching anticodons to mRNA codons to result in a protein.

While there are some aspects of Crick’s hypothesis that did not bear fruit (e.g., the size of the tRNA), much of what he predicted was indeed accurate, even in the absence of experimental evidence identifying those molecules. The tRNAs individually transport amino acids to the ribosome so that proteins can be synthesized. For each tRNA to obtain its cognate amino acid, a cognate aminoacyl-tRNA synthetase (AARS) catalyzes the reaction to attach an amino acid to the tRNA 3ʹ-end, resulting in a “charged” state (Figure 1a,b). An elongation factor protein transports the aminoacylated, or “charged”, tRNA to a free A-site on the ribosome and ensures the accurate association of the correct tRNA anticodon with each mRNA codon. The tRNAs provide the amino acids one by one until the protein synthesis is complete (Figure 1c). In this review, we will focus on tRNA dynamics, folding, and fluctuations, allosteric interactions, and catalytic aminoacylation and editing mechanisms with aminoacyl-tRNA synthetases (Figure 1a,b).

### 1.1. Overview of Family of tRNAs and AARSs

**Figure 2:**
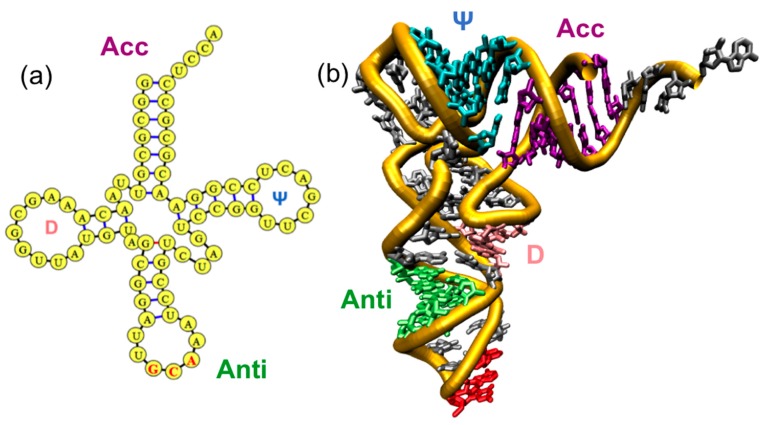
General structure of the tRNA molecule. (**a**) A “cloverleaf” secondary structure of the tRNA molecule with the D (pink), Anti (green; anticodon in red), and TΨC (Ψ) loops (blue) and Acceptor stem (purple); (**b**) The corresponding tertiary structure with the same color scheme.

tRNA molecules are the most well-studied class of RNA molecules because of their small size and universal importance in translation [3,4]. Every organism has at least 20 different tRNA molecules, at least one for each amino acid. Although they vary in sequence significantly, they generally share a conserved “cloverleaf” secondary structure that consists of the D, Anticodon (Anti), and TΨC (Ψ) hairpin loops and an acceptor (Acc) stem (Figure 2a). With few exceptions, tRNAs adopt an “L”-shaped tertiary structure in which the Anti loop and Acc stems are on opposite ends and the D and Ψ loops form the tRNA core [5] (Figure 2b).

The biological function of AARSs is to catalyze the attachment of an amino acid to its cognate tRNA for protein biosynthesis. Despite the conserved chemical reaction, AARS structures, sizes, and amino acid sequences are very diverse. An organism typically has 20 AARSs, although most microorganisms lack AsnRS and/or GlnRS and rely on indirect paths to generate the full complement of aminoacyl-tRNAs. The general requirement that an organism must have a complete set of charged tRNAs to be viable makes AARSs attractive targets for the development of antibiotic or antifungal agents. An example is pseudomonic acid, an antibiotic commercially available as mupirocin [6]. AARSs have been implicated in autoimmune dysfunction, cancer, and cellular regulation pathways that are unrelated to protein synthesis [7]. The enzymes are divided into two classes based on the presence of a Rossmann dinucleotide-binding fold (class I) or an alternative antiparallel β-fold (class II) (Table 1). Class I AARSs are mostly monomeric but can also be monomeric or dimeric. Class II representatives are dimeric and multimeric proteins.

**Table 1:** Table of AARSs by class. LysRS (denoted with a *) can exist as either a class I or class II AARS. AARSs can exist as a monomers (α), homodimers (α_2_), or homo- or hetero-tetramers ((α_2_)_2_/α_4_ or α_2_β_2_).

	Class I	Class II
Group a	ArgRS (α)	GlyRS (α_2_)
	CysRS (α/α_2_)	HisRS (α_2_)
	IleRS (α)	SerRS (α_2_)
	LeuRS (α)	ThrRS (α_2_)
	LysRS * (α/α_2_)	
	MetRS (α/α_2_)	
	ValRS (α)	
Group b	GlnRS (α)	AsnRS (α_2_)
	GluRS (α)	AspRS (α_2_)
		LysRS * (α_2_/(α_2_)_2_)
Group c	TrpRS (α_2_)	AlaRS (α, α_2_, α_4_)
	TyrRS (α)	GlyRS (α_2_β_2_)
		PheRS (α, α_2_β_2_)

Representative high resolution X-ray crystallographic structures have now been determined for all twenty standard AARSs, either alone or in complex with their cognate tRNA (e.g., MetRS [PDB: 1QQT] or CysRS:tRNA^Cys^ [PDB: 1U0B], respectively), as well as for pyrrolysyl-tRNA synthetase (PylRS) and phosphoseryl-tRNA synthetase (SepRS) [8]. Of these, some AARSs have also been crystallized in the presence of substrates, such that the structures represent catalytically competent complexes.

**Figure 3:**
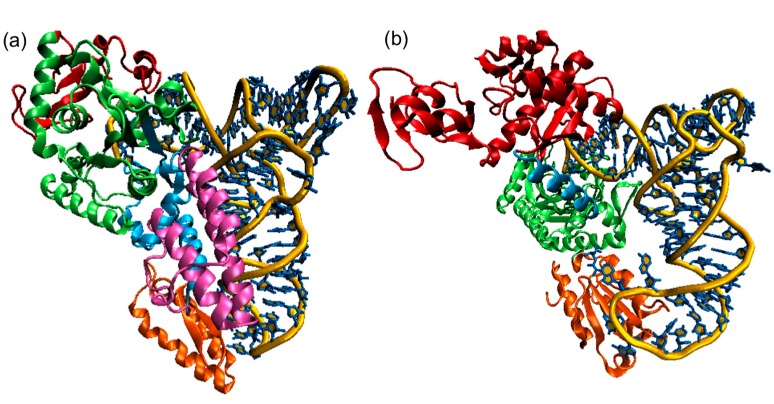
Structures of Class I and Class II AARSs (**a**) *E. coli* CysRS:tRNA^Cys^ complex. The CP domain (red) and Rossmann fold catalytic domain (green), stem contact fold (cyan), helical bundle domain (magenta), and anticodon binding domain (orange) of CysRS are shown in a ribbon diagram; (**b**) A single monomer of the homodimeric *E. coli* ThrRS:tRNA^Thr^ complex. The two N-terminal domains (red), catalytic domain (green), linker (cyan), and anticodon binding domain (orange) of ThrRS are shown in a ribbon diagram. For both structures, the tRNAs are shown in a stick diagram (blue) with a trace of its backbone (yellow).

The AARSs have at least two distinct domains (see Figure 3 for examples of Classes I and II AARSs). The catalytic domain synthesizes aminoacyl adenylate and transfers the amino acid to the cognate tRNA. Another major domain typically contacts the cognate tRNA anticodon (anticodon binding domain). These two major domains mirror the L-shape of the tRNA molecule with its acceptor and anticodon loops, and they are separated by up to 70 Å. Additional polypeptide domains are responsible for dimerization/multimerization, catalytic editing of mischarged tRNA (connective peptide (CP) domain in Class 1a AARSs), assembly of multienzyme complexes, or cellular localization [9].

**Figure 4:**
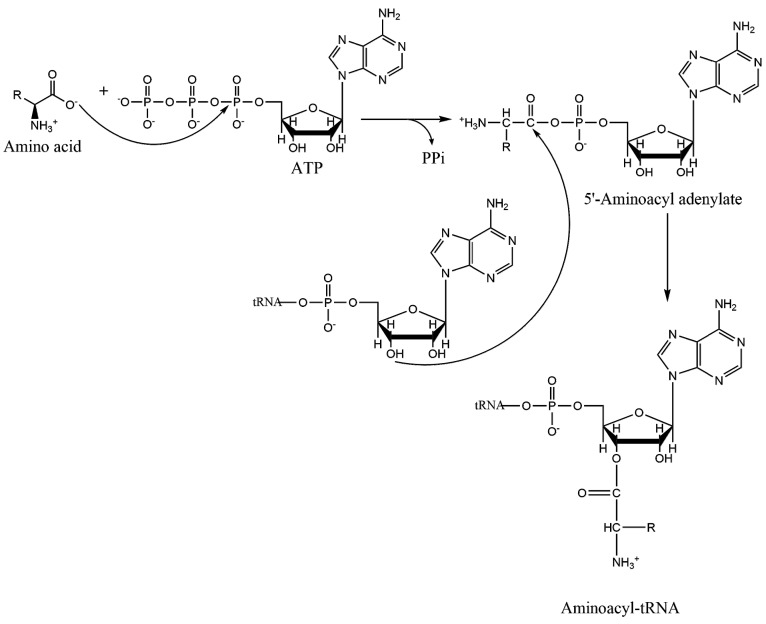
Catalytic mechanism of aminoacylation by aminoacyl-tRNA synthetases. The top row represents the first step in which an aminoacyl adenylate intermediate is formed. In the second step, the “charged” aminoacyl-tRNA is formed. Binding of cognate tRNA is required for adenylate formation for GluRS, GlnRS, ArgRS, and LysRS-1 enzymes. Class II AARSs catalyze nucleophilic attack on the adenylate using the A76 3ʹ-hydroxyl; class I enzymes use the 2ʹ-OH for initial attack before transesterification to the 3ʹ-position.

### 1.2. Mechanism of Aminoacylation Catalytic Reaction

Much of our knowledge about the two chemical steps that occur comes from kinetic analyses. Covalent attachment of an amino acid to its cognate tRNA happens in two steps (Figure 4). An amino acid carboxylate first attacks the alpha phosphate of an ATP, producing an aminoacyl adenylate intermediate. The 2ʹ- or 3ʹ-hydroxyl of the tRNA A76 subsequently displaces the AMP, resulting in a “charged” tRNA attached to its amino acid.

## 2. Biomolecular MD Simulations Approaches

Although very fine experimental techniques such as single molecule experiments can describe molecular features, there is still much to learn about the molecular details of tRNA dynamics and folding and their recognition and allosteric communication networks when in complex with AARSs. A well-established approach is molecular dynamics (MD) simulations, and relatively recent studies using the approach have elucidated the microscopic interactions that contribute to folding, binding, and catalytic mechanisms of tRNAs and AARSs.

MD simulations are designed to be largely consistent with basic physical principles and are amenable to predictions that can be validated by experiments. The 2013 Nobel Prize in Chemistry was awarded to Martin Karplus, Michael Levitt, and Arieh Warshel, who pioneered the MD simulations methodology for bimolecular systems [10,11]. MD simulations evaluate the interactions between macromolecules as a function of the coordinates of their individual substituent particles (e.g., atoms, residues/nucleotides, *etc.*). A molecule’s motion is defined by the potential energy function, which characterizes how the particles interact. The derivatives of the potential energies are numerically calculated to obtain the forces, which are used to solve Newton’s equations of motion, moving the biomolecules in consecutive steps to generate a trajectory [12]. Although chemical bond formation and dissolution cannot be taken into account without quantum mechanics, the classical approximation in MD simulations can describe biomolecular conformational changes with remarkable accuracy.

**Figure 5:**
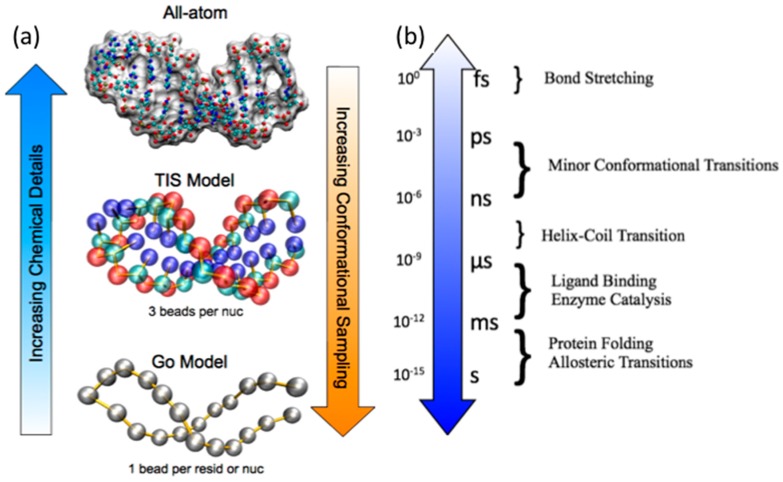
Molecular dynamics approaches at different levels of chemical detail and the associated typical timescales of biomolecular motions. (**a**) The main motivation for developing chemically simplified, coarse-grained approaches is the degree of conformational sampling that results from longer timescales that can be studied; (**b**) The approximate timescales for various biomolecular motions are listed.

While the MD simulation approach is straightforward, simulating relevant-sized protein and RNA biomolecules on timescales (Figure 5b) that can be directly compared with experiments remains a challenge. Protein and RNA biomolecular folding experiments show that these timescales are on the order of 10–1000 μs [13], which is fast given the inherent complexity of the processes [14,15,16,17]. The major bottleneck to MD simulations is the force evaluation, specifically the long-range van der Waals and electrostatic interactions that must be computed for each pair of interacting components.

In the next two sections, we will discuss two classes of MD simulations that have been used for tRNAs and AARSs: Atomistic empirical force field MD simulations and coarse-grained native structure based MD simulations. We realize that most researchers in the tRNA and AARS field may not be well-versed with MD simulations, and the subsequent sections are intended to be a broad overview of basic concepts and techniques, as well as a survey of available resources to encourage non-specialists to perform MD simulations themselves.

### 2.1. Atomistic Empirical Force Field MD Simulations

Empirical force field MD simulations are a class of simulations where biomolecular interactions are based on force field parameters [18]; these were originally intended to study the dynamics of proteins in the native basin. Typically, they represent biomolecules at atomistic resolution, which is computationally demanding and restricted to very small (~50–100 amino acids long), fast-folding (~μs–ms) proteins.

The pairwise additive potential energy function of empirical force field MD simulations usually consists of a large number of parameterized terms, and these parameters are obtained from empirical and quantum mechanical studies of small model compounds. It is assumed that the parameters of smaller model compounds may be combined with others to develop a set of parameters for larger molecules of interest such as proteins or nucleic acids. The set of parameters is called a “force field”, and these have been specifically designed for the simulation of proteins, nucleic acids, and other biomolecules and small organic molecules that can be used in drug design studies. Force fields are designed to be consistent with each other so the study of, for example, protein-RNA complexes can be done readily. Some commonly used empirical force fields include the CHARMM [19], AMBER [20], and GROMOS [21] force fields.

To perform MD simulations using these force fields, a wide variety of software packages can be used that largely differ in the user interface, supported features, and advanced techniques. The underlying MD simulation integrators are largely the same. Some of the most commonly used MD simulation packages include AMBER [22], CHARMM [23], GROMACS [24], LAMMPS [25], and NAMD [26]. These software packages also have large communities of users, available documentations, automatic input setup servers (e.g., CHARMM-GUI [27]), and small molecule parameterization servers (e.g., CHARMM General Force Field (CGenFF) [28] or General AMBER Force Field (GAFF) [29]) to help new users get started. Supercomputing facilities composed of an array of processors with fast interconnects are required as MD simulation algorithms require significant communication and synchronizations between processors to perform the force evaluations.

#### 2.1.1. Addressing Computational Challenges

In general, there are two main computational challenges for MD simulations of biologically relevant biomolecular systems to observe biologically relevant functional events. The first is the large system size of many interesting biomolecules. Very recently, MD simulations of the complete HIV-1 capsid (64 million atoms) was performed using NAMD for 100 ns [30]. The second is the timescale required for the proteins and nucleic acids to perform their biologically relevant motions. Large structural rearrangements or unfolding events are considered very challenging, even for relatively small systems (~100 residues) or small timescales (~μs–ms). The current state of the art is to use advanced computing infrastructures such as the distributed computing Folding@Home approach [31] or the Anton supercomputer [32], which can reach the millisecond timescale for small systems.

However, the average computational biophysicist does not reach the size- and time-scales of the state of the art due to limitations on computational resources. Instead, approximations or advanced sampling techniques can be employed that can reduce computational time, but these approaches typically result in loss of kinetic information. The largest bottleneck of any MD simulation is the force evaluation, in particular the solvent interactions. It is, therefore, desirable to avoid using explicit water if possible without sacrificing accuracy, which may not be reasonable if electrostatic effects are known to be important. Implicit solvent models, such as the Poisson-Boltzmann (PB) [33] and generalized Born (GB) model [34,35], represent the solvent as a continuous medium usually based on the solvent accessible surface area. Implicit solvent model MD simulations have an added benefit of more accurate calculations of the solute-solvent interaction free energies because the entropy calculations from explicit solvent MD simulations might have errors arising from incomplete sampling of solvent calculations that is not an issue for implicit solvent models [36]. The PB and GB models also lack viscosity from water molecules colliding and impeding biomolecular motions. While this increases conformational sampling, which could be seen as a benefit, it also means that kinetic information is lost.

Some commonly used advanced sampling methods include replica exchange [37], metadynamics [38], umbrella sampling [39], and accelerated MD [40], which have been extensively used in protein and nucleic acid systems. Again, these methods increase conformational sampling for more accurate thermodynamics, but it is at the sacrifice of kinetics. We note that the replica exchange, metadynamics, and umbrella sampling approaches are general and can be used for any type of MD simulation, including the native structure based MD simulations we describe in Section 2.2.

Graphics processing units (GPUs) that have been traditionally used for rendering images are now being repurposed for MD simulations [41,42]. The MD simulation algorithm is readily parallelizable, and codes can be recast for the GPU instead. For atomistic empirical force field MD simulations, NAMD [43], AMBER [44], and GROMACS [45] have now been successfully ported to the GPU architecture, as have a number of coarse-grained or general particle dynamics MD simulation codes such as HOOMD-Blue [46], LAMMPS [47], OpenMM [48], and SOP-GPU [49]. The porting of the MD simulation code is often non-trivial and requires the development of new parallel algorithms specifically optimized for the GPU [50]. However, once the technical details are overcome and if the GPU hardware is available, a significant computational performance gain can be obtained in some cases. Since GPUs are separate devices on a computer, each transfer of information between the CPU and the GPU results in a performance penalty hit from the long transfer time. For small systems with very few atoms, it may not be worthwhile to take a performance penalty hit by transferring the simulation information to the GPU device, perform the fast calculation, and transfer the information back. For larger systems, however, a significant computational performance gain is obtained because the speedup in computations is worth more than the information transfer performance penalty [50].

#### 2.1.2. Improving Chemical Details

Finer chemical details can be added to the standard MD simulation approach to more accurately represent biomolecular systems. The partial charges in MD simulations are traditionally represented as static fixed charges that are located at the center of an atom. An atom’s electronic charge distribution can be distorted from its normal shape by introducing an electric field. For example, London dispersion forces introduce a charge polarization so it is no longer centered on the atom. Recently, polarizable force fields have been developed to improve the electronic description of biomolecular environments, and the Drude Oscillator model is becoming the most widely accepted approach [51,52]. In this model, a massless auxiliary charged “Drude particle” is attached to each nucleus by a spring [51,52]. Proteins with charged residues can benefit from polarizable force fields but the largest impact is likely to occur in highly charged systems such as nucleic acids [50]. The implementation of the Drude polarizable force field scales very well on massively distributed supercomputing platforms and takes less than twice as long as a traditional force field [53].

The solution pH is also another important factor that may play an important role in biochemical processes. Traditional MD simulations are performed with fixed protonation states that are often determined at the beginning of each MD simulation. Since X-ray crystallographic structures do not have hydrogen coordinates, the protonation state of a given residue is typically assigned manually assuming a neutral pH or PROPKA can quickly estimate the protonation state of each residue [54] that can be assigned for the duration of an MD simulation. However, the pKa of the amino acids in a protein can significantly change during the course of an MD simulation if the protein if flexible or if the environment of the buried amino acid changes significantly. Constant pH MD simulations allow the protonation states of titratable sites to be determined during the course of the MD simulation at a specified pH [55,56,57,58].

### 2.2. Coarse-Grained Native Structure Based MD Simulations

The question of how biomolecules reach the folded state while discriminating against all other countless alternatives is known as the “protein folding problem”. It has been shown by Cyrus Levinthal in 1969 that protein folding cannot be a random search because the timescales of such a process must be on the order of the age of the universe (*i.e.*, effectively impossible) [59]. Since folding processes are not just a random search, the solution to “Levinthal’s Paradox” is that protein folding must be a directed and biased one, but we must still understand how it occurs and identify the main driving forces involved. The related RNA folding problem has become increasingly appreciated because non-coding RNA molecules also play critical roles in a variety of cellular functions even though they are not templates for translation [60,61]. We now know that non-coding RNA molecules’ functions include catalysis, replication, translational regulation, and ligand binding [62]. The changes in the expression level of non-coding RNAs have been associated with cancer, neurological, cardiovascular, and developmental diseases, and other disorders [63]. Consequently, it is important to determine the mechanisms by which RNA molecules assemble.

Recent work using coarse-grained models similar to those in protein folding strongly suggests that the global folding mechanism of RNA molecules, from both thermodynamic and kinetic viewpoints, is very complex, oftentimes involving parallel folding mechanisms. However, RNA folding mechanisms are not hopelessly complex in that the general order can be accurately predicted based on stabilities of constituent hairpins, at least in simple RNA pseudoknots [64] and tRNAs [65]. To increase simulation timescales, some researchers use coarse-grained MD simulations that still capture folding and binding mechanisms of biologically relevant sized protein and RNA that are simulated at biologically relevant timescales [66,67] (Figure 5). Groups of atoms are represented as a bead or a group of beads whose degrees of freedom that are considered negligible in the overall folding mechanism are excluded, thereby reducing N so that these simulations become feasible to compute.

The native structure based Go-type model is a class of physical coarse-grained MD simulations that has had success in simulating protein and RNA folding mechanisms. It assumes that all attractive interactions contribute to the folded state (“native interactions”) and all other (“nonnative”) interactions are repulsive. Based on the Energy Landscape Theory [15], if the final native structure is known, the Go-type simulation will predict how it gets there. Despite the model’s simplicity, it can reliably predict whether the protein folding mechanism involves an intermediate [68], the folding rates [69], and oftentimes the transition state structures at a residue-level resolution [68]. Furthermore, protein–protein binding and assembly mechanisms [70], as well as RNA folding mechanisms [64,65,71,72,73] have been shown to agree well with experiments, even for coarse-grained variants of the model where an amino acid is represented by a single bead centered at the Cα position of a residue (Go model) or a nucleotide is represented by three beads centered on the base, sugar, and phosphate moieties (Three Interaction Site or TIS model). The long-range interactions of the Go model are the native structure interactions.

The TIS model also includes terms corresponding to sequence dependent base-stacking interactions using the well-known Turner’s Rules and ion concentration-dependent electrostatics for the charged phosphates using the Debye-Hückel potential, which has a simplified distant dependent screening factor. Recent TIS model simulation studies have shown that the telomerase RNA pseudoknot folding mechanism is reproduced with quantitative accuracy and exhibits fast and slow phases with folding rates that fit a biexponential [64]. The folding rates predicted from TIS model MD simulations by Cho *et al.* [64] have been subsequently verified by laser temperature jump perturbation experiments by Ansari and coworkers who measured remarkably similar relaxation times [74].

Native structure based Go-type model MD simulations can also be performed with more detailed representations of protein and RNA. Both proteins and RNA can be given an all-atom representation [75,76]. In addition, the model can be sequence dependent by assigning statistical potential weights to the native interactions [77,78]. In addition, solvent effects can be added by adding a water-mediated interaction term [79]. These coarse-grained MD simulations can readily study the folding and binding mechanisms of proteins or RNAs that are about 200–300 amino acids or nucleotides long.

### 2.3. Coarse-Grained Topological Constraint Based RNA MD Simulations

Recently, Mustoe *et al.* developed a new coarse-grained MD simulation model for RNA dynamics called TOPRNA that restricts the RNA helices across two-way junctions by introducing stereochemical constraints [80]. For RNA folding, since secondary structure generally precedes tertiary structure [81], although notable exceptions exist [64,82,83], Herschlag and coworkers hypothesized that the formation of secondary structure introduces topological constraints that prevent non-native tertiary interactions from forming [84]. As such, basic steric and stereochemical forces can significantly restrict the conformations allowed by helices [85].

In TOPRNA, the RNAs are semi-rigid helices linked by freely rotatable single strands that connect the helices [80]. The secondary structures of the helices are permanently formed and forced to adopt an A-form helical conformation while the single strands are allowed to adopt any conformation that does not violate local bond and angle constraints. As a result, once the secondary structure is formed, the allowed tertiary structure space is exceedingly small. The free energy landscapes restricted by the topological constraints in TOPRNA simulations are similar to the distribution of bulge conformations observed in the PDB [80]. Furthermore, their simulations of the HIV-1 TAR quantitatively reproduce NMR RDC measurements [80].

## 3. tRNA Dynamics

The first MD simulation study of an RNA molecule was of the 76 nucleotide yeast tRNA^Phe^ *in vacuo* (*i.e.*, without solvent) by McCammon and Harvey in 1987 [86]. In their 32 ps MD trajectory, the hydrogen bonds in the secondary structure remained but several important tertiary structures were only transiently observed. Tremendous progress has been made since then due to improvement of force fields and especially the development of particle mesh Ewald (PME) methods that more fully take into account the long-range electrostatic interactions [87]. Stable MD simulations of nucleic acids in the presence of solvents can extend well beyond the microsecond timescale for solvated and neutralized nucleic acid systems [88].

The structural and dynamic properties of RNA molecules are directly influenced by the specific hydration of RNA molecules, and MD simulations can identify hydration sites in which water molecules exhibit long residence times. In their short MD simulations of yeast tRNA^Asp^, Auffinger and Westhof observed that there were few solvent-accessible hydrophilic sites that were occupied by water molecules with long residence times, and these mediated interactions between successive phosphate oxygen atoms that pointed towards the major grove. In contrast, most water molecules rapidly exchanged with the ribose 2ʹ-OH in the minor groove, and the authors suggested that these water molecules would be more readily displaced by drugs that inhibit nucleic acid folding or binding to ligands or proteins [87]. They also observed that a post-transcriptionally modified pseudouridine rigidified loops throughout the tRNA^Asp^ structure through long-lived water-mediated interactions [89]. These loop stabilizations may be required for anticodon discrimination and catalytic activity. More recently, Roh *et al.* [90] used a combination of neutron scattering spectroscopy and MD simulations to characterize a “dynamical transition” that is strongly dependent on hydration. That is, the higher density of water leads to a more flexible tRNA molecule [90].

## 4. tRNA Folding Mechanisms

### 4.1. Brief Overview of Classical and Recent tRNA Folding Experiments

Crothers and coworkers first established the general features of tRNA folding in their seminal thermal denaturation experiments [91,92]. The melting profiles of the tRNAs differed, highlighted by a comparison between tRNA_I_^Tyr^ and tRNA_II_^Tyr^, which came from the same species, code for the same amino acid, and have presumably very similar structures. A general theme arose from their survey of different tRNAs in that they observed multiphasic melting curves under different salt concentrations, suggesting intermediate structure(s) in the folding process. More recently, it was shown experimentally that Na^+^- and Mg^2+^-induced folding of tRNA^Phe^ occurs by at least two transitions that involve secondary structure formation. Temperature-dependent unfolding occurs through distinct sequence-dependent ensembles, and the melting order of the secondary and tertiary structures were not conserved [93]. Furthermore, the exact order of folding depends on the stability of the tRNA hairpins in the presence of the salts, and the folding kinetics can involve a fast and slow phase, suggesting parallel folding mechanisms [94,95,96]. Very recently, Perona and coworkers introduced a new method to track tRNA^Gln^ folding kinetics using the activity of its cognate aminoacyl-tRNA synthetase as a probe. They also observe fast and slow phases derived from a biexponential fit [97] that may also indicate parallel folding mechanisms. Modified nucleotides contribute to tRNA folding in ways that are not fully characterized, in part because of the parallel challenges of isolating native tRNA from cells and synthesizing modified tRNAs [98]. Force field parameters for modified nucleotides have been developed for the AMBER force field [99], but they have not been rigorously tested in a folding mechanism study, for example, to date.

### 4.2. Base Stacking Interactions Are a Main Determinant of Parallel tRNA Folding Mechanisms

Recently, Li *et al.* performed TIS model MD simulations of several *E. coli* tRNA molecules to characterize their folding mechanisms [65]. Even though the tRNA structures they studied are very similar in their lengths and tertiary structures, they observed distinct parallel folding mechanisms. Of particular note was that their sequences were also distinct. For protein folding, it is generally thought that their structures determine their folding mechanisms, although there are some notable exceptions. Since tRNAs generally have well-conserved secondary and tertiary structures, in the TIS model MD simulations of tRNAs, the native contact and electrostatic interactions were largely the same and only the sequence dependent base stacking interactions would differ. Native structure-based [100] and *ab initio* coarse-grained MD simulations [101] also predict intermediate states in the folding of yeast tRNA^Phe^.

**Figure 6:**
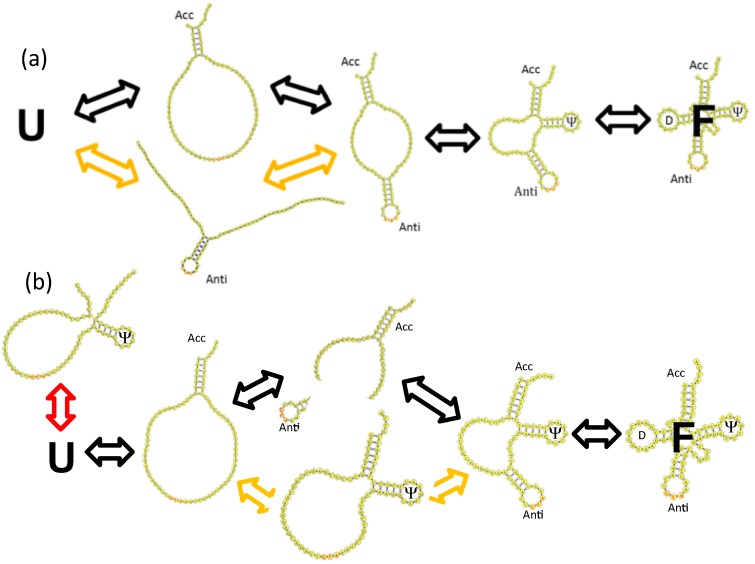
Folding mechanism of tRNAs inferred from TIS model MD simulations. (**a**) Parallel folding mechanism of *E. coli.* tRNA^fMet^; (**b**) Parallel folding mechanism of *E. coli.* tRNA^Tyr^ with backtracking of the Ψ hairpin loop. U and F refer to the unfolded and folded states, respectively. The black arrows represent the dominant mechanism with the lowest free energy barriers, the yellow arrows represent a parallel mechanism with higher free energy barriers, and a red arrow in (**b**) represents a backtracking mechanism.

In their MD simulations, Li *et al.* were able to reproduce the ion concentration dependent melting profiles of Crothers and coworkers, and they projected the free energy to the tRNA hairpins and stem and observed parallel folding mechanisms that could be predicted by the stabilities of their individual hairpins and stem (Figure 6a) [65]. A possible explanation for the fast *vs.* slow folding rates could be the parallel folding mechanisms they observed in their MD simulations.

### 4.3. Backtracking Mechanisms Partitions Fast vs. Slow Folding

Another possible nonexclusive explanation for the disparity in the folding rates is the presence of a premature nonproductive off-pathway intermediate in the folding mechanism. In MD simulations of the tRNAs, Li *et al.* also observed that the unstable Ψ hairpin loop could form very early in the folding mechanism but then had to completely unfold, or “backtrack” in order for the folding mechanism to proceed (Figure 6b). Clementi *et al.* first predicted the backtracking mechanism in the context of protein folding [68].

Interleukin-1β is a β-trefoil containing protein that is known to have a very slow folding rate. Gosavi *et al.* observed in their Go-model MD simulations of Interleukin-1β that a β-bulge introduces topological frustration in the funneled Go model folding energy landscape [102]. Since not all β-trefoil containing proteins are slow folders, they replaced the β-bulge with an analogous loop from the structurally and functionally related Interleukin-1 receptor agonist and observed faster folding. They postulated that the presence of the β-bulge slows down its folding to inhibit its function so that it is not always acting under optimal conditions [103].

Hills and Brooks observing backtracking in their Go model MD simulations of CheY, NtrC, and Spo0F [104]. In a very interesting study, Matthews, Brooks, and coworkers experimentally verified the backtracking mechanism prediction by synthesizing CheY permutants to alter the chain connectivity and minimize the chain entropy such that an off-pathway intermediate can be destabilized to promote folding [105]. To date, the backtracking mechanism for some tRNAs predicted by Li *et al.* is the first for nucleic acids, but they have yet to be verified experimentally. However, it could be done, in principle, by synthesizing a permutant in which the acceptor stem is closed but a break is introduced in the Ψ hairpin loop so that it cannot prematurely form. The expected outcome is that a slow folding phase would be eliminated from the kinetics.

### 4.4. Topological Constraints in tRNA Tertiary Structure

Very recently, Mustoe *et al.* used their coarse-grained TOPRNA model MD simulations with topological constraints for yeast tRNA^Phe^ and modeled changes to the variable arm. The topological constraints introduced by the secondary structure formation prevent non-native conformations from forming and optimizing folding for the native tertiary structure. The reduction of the entropic penalty for forming the native tertiary structure increases folding cooperativity. They also explored how variable loops could actually become base-paired stems, as observed in class II tRNAs, which have elongated variable loops. In their TOPRNA model MD simulations, variable loops that are longer than 5-nts destabilized the native tertiary structure, suggesting an evolutionary pressure to restrict loop size [106]. In a separate study by Mustoe *et al.*, TOPRNA model MD simulations were performed for human mitochondrial tRNA^Ser (UCN)^ to understand how its non-canonical secondary structure still formed a canonical tertiary structure. They observed that non-canonical tRNAs have increased topological constraints that compensate for the loss of tertiary structure. A pathogenic mutation lowered the melting temperature and increased the folding free energy as measured by UV melting experiments, and the same was observed in their MD simulations in quantitative agreement [107].

## 5. MD Simulations of Aminoacyl-tRNA Synthetases

### 5.1. Brief Overview of MD Simulations of AARSs

The earliest MD simulations of an aminoacyl-tRNA synthetase were performed by Karplus and coworkers in 1994 to determine the binding free energy difference of a tyrosine substrate between the wild type *Bacillus stearothermophilus* TyrRS and its Y169F mutant [108]. Since the enzyme was too large at the time to be computationally tractable, they employed a “stochastic boundary” approach by isolating an approximately spherical region around the area of interest and removing the rest of the enzyme from their MD simulations. A buffer region around the spherical region was harmonically constrained to account for missing atoms. Their production run MD simulation time for their truncated free and bound systems was 540 ps. Even with this simplified system, the calculated free energy change was in good agreement with the experimentally measured value and within statistical error [108].

In the 20 years since that seminal study, the field has expanded with increased access to computing resources and widely available software packages that enable MD simulations on much larger structures. The increased accuracy of atomic force fields also allows far more accurate simulations. In the past decade, MD simulations of aminoacyl-tRNA synthetases are now generally performed for their full structures on the 1–100 ns timescale (Table 2 and Table 3). Many research groups now focus on MD simulations that characterize flexibility, long-range allosteric communication networks, or catalytic mechanisms that would not have been possible to study with a truncated system such as the one originally used by Karplus and coworkers.

**Table 2:** MD Simulations of Class I AARSs.

AARS	Ligands	Starting Structure(s)	Time	Reference
CysRS	+tRNA^Cys^:Cys-AMP (modeled) +Cys-AMP (modeled)	1LI5 and models	10 ns	Ghosh *et al.*, JBC, 2011 [109]
GlnRS	+tRNA^Gln^ (modeled)	4H3S and models	70 ns	Grant *et al.*, JMB, 2013 [110]
GlnRS	+tRNA^Gln^	1GTR, 1EXD and models	6.5 ns	Yamasaki *et al.*, Biophys. J, 2007 [111]
GluRS	+tRNA^Glu^:Glutamol-AMP	1N78	20 ns	Pyrkosz *et al.*, JMB, 2010 [112]
GluRS	+tRNA^Glu^:Glu-AMP	1N78	20 ns	Sethi *et al.*, PNAS, 2009 [113]
LeuRS		CP domains from 3PZ0, 3PZ6	20 ns	Liu *et al.*, Biochem. J, 2011 [114]
LeuRS	+tRNA^Leu^:Leu-AMP (modeled)	1WZ2, 2V0C	20 ns	Sethi *et al.*, PNAS, 2009 [113]
LeuRS		1H3N	55 ns	Strom *et al.*, J. Mol. Model., 2014 [115]
LeuRS	+Val-tRNA^Leu^ (modeled)	2BYT, 10BC and models	1 ns	Hagiwara *et al.*, FEBS, 2009 [116]
MetRS	tRNA^Met^:Met-AMP	2CSX, 2CT8 and models	10 ns	Ghosh *et al.*, PNAS, 2007 [117]
MetRS		1QQT	12 ns	Budiman *et al.*, Proteins, 2007 [118]
MetRS	+Met, +ATP, +Met-AMP, +tRNA:MetAMP (modeled)	1QQT, 1F4L, 1PFY and models	10 ns	Ghosh *et al.*, Biochem., 2008 [119]
MetRS		1QQT	30 ns	Strom *et al.*, J. Mol. Model., 2014 [115]
TrpRS	+Trp-AMP, +tRNA^Trp^:Trp-AMP (modeled)	2DR2, 1R6U and models	5 ns	Bhattacharyya *et al.*, Proteins, 2008 [120]
TrpRS	+ ATP, + Trp, +ATP:Trp, +ATP:Mg, +ATP:Trp:Mg	1MAW, 1MB2, 1MAU, 1M83, 1I6L	5 ns	Kapustina *et al.*, JMB, 2006 [121]
TyrRS	+Tyr, +ATP, +Tyr-AMP, +inhibitor	1JIL, 4TS1, 1H3E, 3TS1, 1I6K and models	12 ns	Li *et al.*, Eur. Biophys. J., 2008 [122]
TyrRS	Tyr	4TS1	540 ps	Lau *et al.*, JMB, 1994 [108]
TyrRS	+Tyr, +Tyr:ATP, +Tyr-AMP	2JAN, 1X8X, 1H3E, 1VBM and models	100 ns	Mykuliak *et al.*, Eur. Biophys. J., 2014 [123]
TyrRS		Assembled N and C domains from 1N3L and 1NTG	100 ns	Savytskyi *et al.*, J. Mol. Recognit., 2013 [124]
ValRS	+tRNA^Val^:Val-AMP, +tRNA^Val^:ThrAMP (modeled)	1GAX and models	10 ns	Li *et al.*, J. Mol. Model., 2011 [125]
ValRS	+editing substrates (modeled)	1WK9 (CP domain), 1GAX (ValRS + tRNA) and models	2 ns for full 5 ns for CP	Bharatham *et al.*, Biophys. Chem., 2009 [126]

**Table 3:** MD Simulations of Class II AARSs.

AARS	Ligands	Starting Structure(s)	Time	Reference
AspRS	+Asp:ATP (modeled), + Asn:ATP (modeled)	1IL2, 1COA and models	500 ps	Thompson *et al.*, Chem. Bio. Chem., 2006 [127]
AspRS	+Asp:ATP (modeled), +Asn:ATP (modeled)	1IL2, 1COA and models	0.5–3 ns	Thompson *et al.*, JBC, 2006 [128]
AspRS	-	1ASZ and models	5 ns	Ul-Haq *et al.*, J. Mol. Graph. Model., 2010 [129]
AspRS	+Asp, +Asn (modeled)	1C0Z	300 ps	Archontis *et al.*, JMB, 2001 [130]
AsnRS	+Asn-AMP, +Asp-AMP (modeled)	*T. thermophilus* AsnRS	4 ns	Polydorides *et al.*, Proteins, 2011 [131]
HisRS	+His-AMP, +His (modeled), +HisOH (modeled)	1KMM, 1KMN and modeled variants	600 ps	Arnez *et al.*, Proteins, 1998 [132]
LysRS (LysU)	+Lys:AMPPCP	1E22, dimer modeled	1 ns	Hughes *et al.*, BMC Struct. Biol., 2003 [133]
LysRS (LysU)	+Lys:AMPPCP	1E22, dimer modeled	520 ps	Hughes *et al.*, Proteins, 2006 [134]
ProRS	+Pro-AMP (modeled)	2J3M	30 ns	Strom *et al.*, J. Mol. Model., 2014 [115]
ProRS	-	2J3M and modeled variants	12 ns	Sanford *et al.*, Biochemistry, 2012 [135]
SerRS	+tRNA^Ser^	3W3S and modeled dimer	2 ns	Dutta *et al.*, J. Phys. Chem. B, 2015 [136]
ThrRS	+tRNA^Thr^:Thr-AMP (modeled)	1QF6	15 ns	Bushnell *et al.*, J. Phys. Chem. B, 2012 [137]

However, there remain some significant challenges for performing MD simulations of AARSs. While there are a plethora of high-resolution X-ray crystallographic structures of these enzymes, in some systems AARS structures do not contain its cognate tRNA or other structures representing steps along the catalytic pathway have not been resolved. As such, many groups have modeled the appropriate structures to carry out their MD simulation studies. Even if the structures were readily available or could be reasonably modeled, despite advances in computer hardware and software, the size of AARSs limits the timescales of simulations that one can perform. Many of the MD simulations of AARSs performed to date span far less than 100 ns (Table 2 and Table 3), which would be the minimum time required even for a simple helix-coil transition (Figure 5b). As such, induced fit structural changes do not occur in these short MD simulations, although fluctuations characterized by high RMSF can be observed in flexible regions. In addition, traditional MD simulations cannot involve chemical bond formation or breaking and have fixed protonation states, which limits the studies of chemical reactions that are required for AARS enzymatic function. Rigorous direct studies of those phenomena require quantum mechanical (QM) calculations that are often done for static structures to infer the electronic structural environment.

The questions AARS researchers have sought to answer using MD simulations are not different from those pursued experimentally: what are the protein and tRNA contributors to recognition and catalysis, and how do the successive steps of substrate binding, amino acid activation, tRNA aminoacylation, and product proof-reading proceed? In the following sections, we survey several recent MD simulation studies to highlight the types of information that can be gleaned from MD simulation studies. In particular, we will focus on AARS MD simulation studies that characterize (1) their allosteric communication pathways between the catalytic and anticodon binding domains; (2) their functional and structural roles of editing domains; and (3) their functional catalytic mechanisms.

### 5.2. Flexibility and Allosteric Communication Networks in AARSs

Proteins in general are dynamic macromolecules, opening active sites to allow substrate binding and product release or responding to allosteric modulator binding. Even for the *Bacillus stearothermophilus* TyrRS, the first AARS structure to be crystallized [138], it was recognized just one year after the publication of its crystal structure that the observed disorder indicated the presence of enzyme flexibility [139]. For protein:RNA complexes in particular, one or both of the macromolecules often exhibits conformational change upon binding [140].

Even in the absence of cognate tRNA, MD simulations using apo-enzyme crystal coordinates can provide information on the inherent flexibility of a protein and which structures might be accessed by low-energy transitions. For example, Budiman *et al.* performed 12 ns MD simulations on monomeric *E. coli* MetRS and observed high mobility of the Zn-binding CP (also CP1 in some literature) domain and class I-conserved KMSKS and rotation of the anticodon-binding domain relative to the catalytic domain [118]. These observations are consistent with the known contribution of KMSKS flexibility to methionyladenylate formation [141] and the CP domain disorder and anticodon-binding domain rotation in the *A. aeolicus* MetRS:tRNA^Met^ co-crystal structure [142].

In the absence of complete crystal structures, all-atom models can be constructed from fragment structures, as in the case of human TyrRS [124]. Multiple 100 ns simulations extended a previous coarse-grained analysis and probed the contacts between catalytic N-terminal domain and EMAP II-like C-terminal domain [143]. Domain interface contacts were consistent with the cytokine activities of TyrRS fragments being masked in the full-length polypeptide [144].

Potential drug target *M. tuberculosis* TyrRS (Mt-TyrRS) has been crystallized in various substrate-free and small substrate-bound arrangements. In an unusually computationally rigorous study, Mykuliak *et al.* performed 3 trajectories of CHARMM27 and AMBER ff99SB-ILDN 100 ns MD simulations for several Mt-TyrRS complexes they modeled [123]. They probed the orientation of the KFGKS (KMSKS-like motif) loop as a function of substrate binding [123]. Simulations revealed short β-sheets forming transiently as the loop closed over the enzyme active site; substitutions that prevented β-sheets formation kept the loop in an open conformation inconsistent with adenylate formation. The corresponding *S. aureus* TyrRS loop is also highly mobile [122], as is the KMSKS loop of *E. coli* MetRS [118]. *In silico* substitution of the highly conserved Trp-461 residue with Ala reduced loop mobility in the MetRS system, consistent with the 10^5^-fold loss of catalytic activity seen for this variant experimentally [145].

As might be expected, polypeptide domains connected by flexible linkers exhibit significant structural rearrangement upon substrate binding; these conformational states can be captured in nanosecond-scale simulations. The CP domain inserted between halves of the Rossmann fold nucleotide binding motif of class I AARSs is dynamic to the point of being disordered in some crystal structures, and MD simulations are consistent with high mobility of this domain in *T. thermophilus* LeuRS [115], and *E. coli* MetRS [115,118]. The N-terminal helical subdomain of yeast GlnRS is also highly mobile [110], as is the editing domain of *Enterococcus faecium* ProRS [115].

In addition to investigating nanosecond scale dynamics, there is also the question of how tRNA binding contributes to catalysis. This is primarily anticodon-triggered aminoacylation, but can also include D-loop triggered post-transfer editing. Vishveshwara and coworkers have applied network analyses to MD simulations such that residues making a direct path (or paths) from can be identified [109,117,120]. One challenge is validating such residues along a path experimentally; presumably there is built-in redundancy in critical biological systems, so it wouldn’t be surprising to find that any one substitution does not eliminate communication.

### 5.3. Conformational Flexibility of tRNA upon Binding to AARSs

Nucleic acids are also highly flexible and can undergo structural rearrangements upon binding to their protein partners. For example, tRNAs aminoacylated by monomeric class I enzymes adopt a unwound or “hairpinned” conformation to access the activated cognate amino acid in the enzyme active site [146,147,148]. Acceptor end distortion is necessary because of the way that the two classes of AARSs bind their cognate tRNAs: class I enzymes bind on the acceptor stem minor groove side, while class II enzymes approach from the major groove side [149]. In the absence of unwinding, tRNAs aminoacylated by monomeric class I enzymes would bypass the active site altogether. (Class Ic enzymes TyrRS and TrpRS are obligate dimers and their tRNAs exhibit cross-subunit binding with major groove recognition [149].)

**Figure 7:**
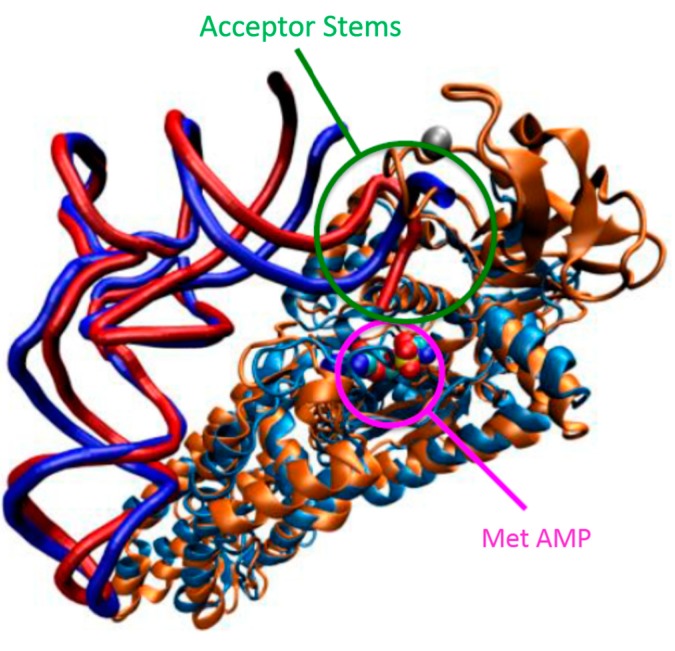
Structure of modeled *E. coli* MetRS:tRNA^fMet^ complex with hairpinned and extended tRNA 3ʹ-end. The *A. aeolicus* MetRS:tRNA^Met^ complex (light blue and dark blue) is superimposed with the *E. coli* MetRS structure (orange) such that the MetRS structures are aligned. The *E. coli* tRNA^fMet^ (red) with a hairpinned 3ʹ-end is structurally aligned to the *A. aeolicus* tRNA^Met^ structure to complete the modeled *E. coli* MetRS:tRNA^fMet^ complex. The Zinc (silver) and Met-AMP are shown in space-filled representation. Note the proximity of the tRNA^fMet^ hairpinned 3ʹ-end to the Met-AMP, the substrate for the aminoacylation reaction.

Of the class I AARS:tRNA crystal structures determined, some display a tRNA acceptor stem that is folded back toward the catalytic site, while others exhibit an extended helix or show some disorder of the terminal nucleotides. The variety of acceptor stem orientations identified by crystallographic studies demonstrates the deformability of the tRNA 3ʹ-end. For example the yeast ArgRS:tRNA^Arg^ structure has a fully hairpinned 3ʹ-end [150], indicative of a catalytically competent complex. In contrast, the *Aquifex aeolicus* MetRS:tRNA^Met^ complex is disordered at the terminal two nucleotides but the remainder of the acceptor stem appears to maintain an extended A-form helix; the enzyme’s CP domain is also disordered, demonstrating the inherent mobility presumably necessary for efficient catalysis [118]. Currently, the structure of the *E. coli* MetRS:tRNA^Met^ complex is absent in the field, so Ghosh and Vishveshwara modeled it based on the structurally homologous *A. aeolicus* complex and performed subsequent MD simulations on their modeled structures [117]. Interestingly, if the tRNA^Met^ is fully extended, it would clash with the CP domain that is present and fully structured in the *E. coli* structure, even if the CP domain were to make minor rotations [118]. Indeed, the RMSD of the modeled complex remained unequilibrated during their 10 ns trajectory, and reached as high as 6.4 Å [117]. However, a hairpinned structure would position the 3ʹ-end in the active site (Figure 7).

### 5.4. Catalytic Aminoacylation Mechanisms of AARSs

Even though MD simulations cannot describe chemical reactions (*i.e.*, chemical bond formation and breaking), they can still inform regarding the active site chemical environment to identify residues that are most likely to participate in catalysis. The tRNA aminoacylation mechanism in AARSs occurs in two half reactions (Figure 4): (1) condensation of a cognate amino acid with ATP to yield an aminoacyl adenylate; and (2) transfer of the aminoacyl group to the cognate tRNA’s A76 ribose at the 2ʹ- or 3ʹ-position.

Aminoacylation requires a base within the active site that deprotonates the target A76 ribose hydroxyl to enhance the nucleophilicity of the hydroxyl oxygen and facilitate its attack at the carbonyl carbon of the adenylate aminoacyl group. The identity of the catalytically relevant Brønsted base is sometimes unclear because the active site pK_a_s typically fluctuate due to the local protein environment, and it remains a challenge to unambiguously identify them. For example, active site histidine substitutions have been shown to significantly reduce the reaction rate [151]. AARS MD simulations with the neutral and protonated forms of the putative active site histidines [137] or quantum mechanical electrostatic potential calculations of the active site residues can determine the feasibility of the residue to act as a mechanistic base. It is also possible that AARSs lack any specific proton-accepting residue, and it has been computationally predicted based on quantum mechanical density functional theory calculations that the nonbridging phosphate oxygen of the aminoacyl adenylate could act as a general base [152]. In a particularly computationally intensive MD simulation study of *Thermus thermophilus* GluRS, Pyrkosz *et al.* showed that a conserved Glu is the likely general base despite proximal histidines that were buried and had pK_a_s predicted by PROPKA that made them neutral [112]. Of course, active site pK_a_s can change significantly during an MD simulation, and Constant pH MD simulations would allow the protonation state to change as the active structure environment changes. Constant pH MD simulations have not been performed for AARSs to date.

### 5.5. Functional and Structural Roles of Editing Domains

It is now well established through decades of experimental and theoretical studies that AARSs have adapted mechanisms to ensure that protein synthesis maintains an exceptionally high fidelity [153]. For tRNA aminoacylation, errors can arise from mistakes in the amino acid activation (pre-transfer) or charging (post-transfer) step [154]. However, in earlier times, it was not obvious that aminoacylation would be as accurate as it is: Linus Pauling predicted in 1958 based on the hydrophobic interaction energies of isoleucine and valine that proteins would be unable to distinguish between these structurally similar amino acids with a sufficiently low error rate [155]. In 1977, Alan Fersht proposed a “two stage editing mechanism” to account for the unusually high fidelity in AARSs. In this model, a misactivated aminoacyl adenylate that is produced in the first major (pre-transfer) step of the aminoacylation reaction is hydrolytically cleared, and a second (post-transfer) step removes any mischarged tRNA by cleaving the incorrect amino acid [156,157].

Class I enzymes LeuRS, IleRS, and ValRS and class II enzymes ThrRS, ProRS, AlaRS, and PheRS have expanded polypeptide modules that function as hydrolytic editing sites for mis-aminoacylated tRNAs [158]. The tRNAs for these editing synthetases must translocate between the synthetic and editing catalytic sites, with the tRNA 3ʹ-end adopting first an unwound structure for amino acid transfer, then an extended conformation for proofreading [159]. For example, *E. coli* LeuRS undergoes significant conformational changes during the catalytic cycle of tRNA binding, aminoacylation, and proof-reading [160]. For these editing synthetases, it is now generally accepted that such post-transfer editing is the dominant contributor to aminoacylation accuracy for all but IleRS [161].

Editing mechanisms have been studied experimentally by directly measuring amino acid release from mischarged tRNA (post-transfer editing) or by aminoacyl-adenylate synthesis and hydrolysis (pre-transfer editing; the relative contributions of the two paths can be determined with enzyme variants lacking efficient post-transfer editing [157,162]. Additionally, the hydrolytic editing activity of some AARSs has been a target for MD simulation analysis. For *T. thermophilus* ValRS, simulations suggested that near-cognate *vs.* cognate adenylate substrates have altered binding affinities [126] and lead to different overall protein mobility [122]. A similar result was observed for the post-transfer aminoacyl-tRNA substrates as well. Several questions remain concerning AARS editing that can be studied using MD approaches. For example, what drives the kinetic partitioning between tRNA *vs.* water attack on the aminoacyl-adenylate in the synthetic active site? What drives conformational changes in tRNA and AARS that triffer relocation of aa-tRNA from synthetic to editing active sites?

In addition to their editing catalytic function, the presence of an editing domain can have a significant impact on aminoacylation function. Using kinetic experiments and MD simulations, Sanford *et al.* deleted an editing domain from *E. coli* ProRS, which resulted in reduced catalytic efficiency due to coupled motion observed in their MD simulations between the editing domain and catalytically important residues that facilitate proline binding [135]. Some AARSs such as *E. coli* MetRS have retained a CP domain that does not have an editing biological function.

## 6. Conclusions

Since the first predictions of both tRNA and aminoacyl-tRNA synthetases, these macromolecules have been subjected to decades of extensive experimental studies largely due to their fundamental roles in protein synthesis. More recent MD simulations are providing increasingly finer details to enhance our mechanistic understanding of these enzymes or make predictions that can be verified by experiments.

In the case of tRNA, a relatively small nucleic acid, advances in MD simulations allow greater accuracy and longer timescale description of their dynamics and folding mechanisms. In particular, empirical force fields have improved in their accuracy to better describe long-range interactions, especially electrostatic PME and other general force field improvements that now allow microsecond timescale MD simulations of tRNA dynamics. However, coarse-grained MD simulation approaches are already providing new insights and predictions for folding mechanisms of tRNA that has not yet been performed with empirical force field MD simulations. Recent studies have shown that base-stacking interactions are the main determinant for the tRNA secondary structure folding, and those secondary structural elements have topological constraints that bias tertiary structure formation towards their native structure while largely excluding nonnative ones. Some tRNAs are predicted by coarse-grained MD simulations to undergo a so-called backtracking mechanism that has been observed and verified in proteins but has yet to be experimentally validated, although it is fairly straightforward to do so as we described above.

Even though high-resolution X-ray crystallographic structures exist for at least one of every type of AARS, structures from many important steps in tRNA recognition, aminoacylation catalysis, and editing are not available for any single AARS. MD simulations using modeled structures of these complexes continue to provide detailed analyses of AARS:tRNA dynamics that demonstrate how flexibility of both AARS and tRNA play fundamental roles in their biological functions. In particular, the identification of allosteric communication networks may require longer timescale MD simulations than currently performed because conformational changes in the AARS require MD simulations on the 100’s of nanosecond timescale. While many of the current analyses in the field have been focused on communication pathways across AARSs, the tRNAs likely play important allosteric roles as well. Further complicating the problem is that AARSs likely evolved redundant allosteric communication networks that remain difficult to experimentally validate. In addition, the identification of critical active site residues for both the aminoacylation and editing mechanisms will likely require very detailed representations of AARS:tRNA complexes.

However, recent advances in computations have also made possible many different types of MD simulations that have not previously been applied to AARSs in particular. Current state of the art high performance computing is now amenable for MD simulations of large systems such as AARS:tRNA complexes. New GPU architectures and successfully ported codes can probably benefit MD simulations of tRNA but the largest speedup will be seen in much larger AARS:tRNA complexes. In addition to these computational hardware advances, advances in MD simulation approaches allow more detailed MD simulations such as polarized force fields and Constant pH MD simulations that can provide more accurate representation of aminoacylation and editing mechanisms for AARSs. In addition, coarse-grained MD simulations can in principle be adapted for the study of protein-RNA complexes such as AARS:tRNA complexes; these will provide a better understanding of the tRNA recognition by AARSs that still remain a challenge. As the finer details of AARS catalysis and editing functions become better understood, their inhibitors can be designed and refined through computational approaches, and the small molecule parameterization servers can enable MD simulations to evaluate their interactions with AARSs. Although all of these approaches are very natural next steps in the field, they have yet to be performed.
